# Next-Generation Sequencing Analysis of Root Canal Microbiota Associated with a Severe Endodontic-Periodontal Lesion

**DOI:** 10.3390/diagnostics11081461

**Published:** 2021-08-12

**Authors:** Alessio Buonavoglia, Gianvito Lanave, Michele Camero, Marialaura Corrente, Antonio Parisi, Vito Martella, Carlo Prati

**Affiliations:** 1 Endodontic Clinical Section, Dental School, Department of Biomedical and NeuroMotor Sciences, University of Bologna, 40125 Bologna, Italy; alessio.buonavoglia85@gmail.com (A.B.); carlo.prati@unibo.it (C.P.); 2 Department of Veterinary Medicine, University of Bari, 70010 Bari, Italy; gianvito.lanave@uniba.it (G.L.); michele.camero@uniba.it (M.C.); vito.martella@uniba.it (V.M.); 3 Experimental Zooprophylactic Institute of Puglia and Basilicata Section of Putignano, Contrada San Pietro Piturno, Putignano, 70017 Bari, Italy; antonio.parisi@izspb.it

**Keywords:** molecular diagnostics, next-generation sequencing, endodontic-periodontal lesion, oral bacteria

## Abstract

A patient with an unusual endo-periodontal lesion, without coronal decay or damage, likely caused by a deep periodontal lesion with subsequent endodontic bacterial migration, required medical care. Next-generation sequencing (NGS) was used to assess the endodontic microbiota in vestibular and palatal canals after tooth extraction, evidencing a predominant population (Fusobacterium nucleatum) in one endodontic canal, and a mixed bacterial population with six major populations almost equally distributed in the other endodontic canal (*F. nucleatum, Porphyromonas gingivalis*, *P. endodontis, Parvimonas, Peptostreptococcus stomatis, Prevotella multiformis*). These data could suggest different, separated ecologic niches in the same endodontic system, with potentially different pathogenicity levels, clinical manifestations and prognoses for every single canal of the same tooth.

## 1. Introduction

Tooth and periodontal tissues can be affected by bacterial infectious diseases such as caries, pulpal and periapical pathologies and periodontitis [1,2].

Although periodontal and endodontic tissues seem to be distinct, there is a strict anatomical correlation between the lateral and accessory canals, apical foramen and dentinal tubules, where bacteria may migrate from one tissue to another [3]. Furthermore, bacterial biofilms associated with endodontic or periodontal infections are similar, confirming these pathways of migration [4].

Endo-periodontal lesions (EPLs) are bacterial infectious diseases that together affect periodontal and endodontic tissues of the same tooth, with periodontal tissue damage and pulp inflammation/necrosis, defined by a pathological communication between the pulpal and periodontal tissues [5].

Despite the bacterial etiology, tissue damage and inflammation are sustained and enhanced by the inflammatory host response with inflammatory mediators such as Galectin-3 (Gal-3), the Nod-like receptor family pyrin domain-containing 3 (NLRP3) inflammasome and Interleukine-1. In particular, Gal-3 is implicated in the regulation of inflammation, as demonstrated in similar disease models [6,7,8].

A pathologic communication between these tissues can occur by a carious or traumatic lesion that affects the pulp and, secondarily, the periodontium between the apical foramen, accessory canals and dentinal tubules. On the other hand, a massive periodontal destruction can secondarily affect the root canal system, with dissemination of the inflammation, which can result in pulp necrosis; the concomitant presence of different and independent pathologies may represent another possibility, and it may be associated with their advancement [9].

Vertical root fractures, cracks and root damage may also serve as a “bridge” for pulp/periodontal contamination [10]. Root planning and scaling may result in removal of the root cementum with exposure of dentinal tubules and rupture of the neurovascular bundle in the lateral canals, leading to pulp alterations [11].

The most common signs and symptoms associated with a tooth affected by EPLs are deep periodontal pockets reaching or close to the apex with a periodontal probing depth (PD) of ≥5 mm and a clinical attachment level (CAL) of ≥3 mm, and a negative or altered response to pulp vitality tests, including spontaneous pain history [5,9].

Other possible signs and symptoms are possible, such as bone resorption in the apical or furcation region, alveolar bone loss, spontaneous pain or pain on palpation and percussion, purulent exudate, tooth mobility, sinus tract, discoloration of the crown due to pulp necrosis and red and swollen gums from gingival inflammation with bleeding on probing [12].

EPLs are often a diagnostic challenge for clinicians, complicated by the fact that these diseases are frequently viewed as independent entities, with a difficult management of both components and uncertain prognosis [11].

In 2018, the American Academy of Periodontology (AAP) published a new updated classification for EPLs, which are distinguished primarily on the basis of the presence of root damage and, secondarily, on the basis of the number and aspect (narrow/wide) of periodontal pockets that affect the tooth surface [9].

Several authors reported a great similarity between the microbiota found in the root canals and periodontal pockets [13,14,15,16]. In general, the predominant bacteria are fastidious pathogens and strict anaerobes, poorly or not cultivatable in vitro, and microbiological investigations rely on metagenomic techniques [17].

Recently, culture-independent techniques, such as next-generation sequencing (NGS), have expanded the knowledge on the oral and dental microbiome [18]. Several unrecognized species have been found, with the possibility to explore the pathogenic role and the biodiversity even across different anatomical sites [15].

By NGS analysis, the most frequently detected bacterial species in the endodontic space are Enterococcus faecalis, Parvimonas micra, Mogibacterium timidum, Filifactor alocis and Fretibacterium fastidiosum, whilst P. micra, E. faecalis, Streptococcus constellatus, Eubacterium brachy, Tannerella forsythia and F. alocis represent the most frequently detected organisms in the periodontal space [15].

The following case report describes an unusual Grade 3 EPL with root damage. Coronal decay was not detectable, and microbiological investigations on the endodontic microbiota were performed with NGS after tooth extraction.

## 2. Materials and Methods

### 2.1. Case Presentation

A 45-year-old male attended a private dental office in Bari (Italy), with the chief complaints of periodic pain, swelling and bad taste from the right maxillary posterior region. The patient reported no traumatic accidents in this area, but he reported a previous dental extraction (tooth 1.8.) a few years ago for the same reasons. The patient referred to a previous implant-prosthetic rehabilitation with two single implants for upper lateral incisor agenesis, which were subsequently removed for periimplantitis. His past medical history was not contributory (ASA 1) [19], and he referred only to sporadic allergic events of unknown origin with diffuse erythema on the entire body, and intestinal dysfunction.

**Figure 1:**
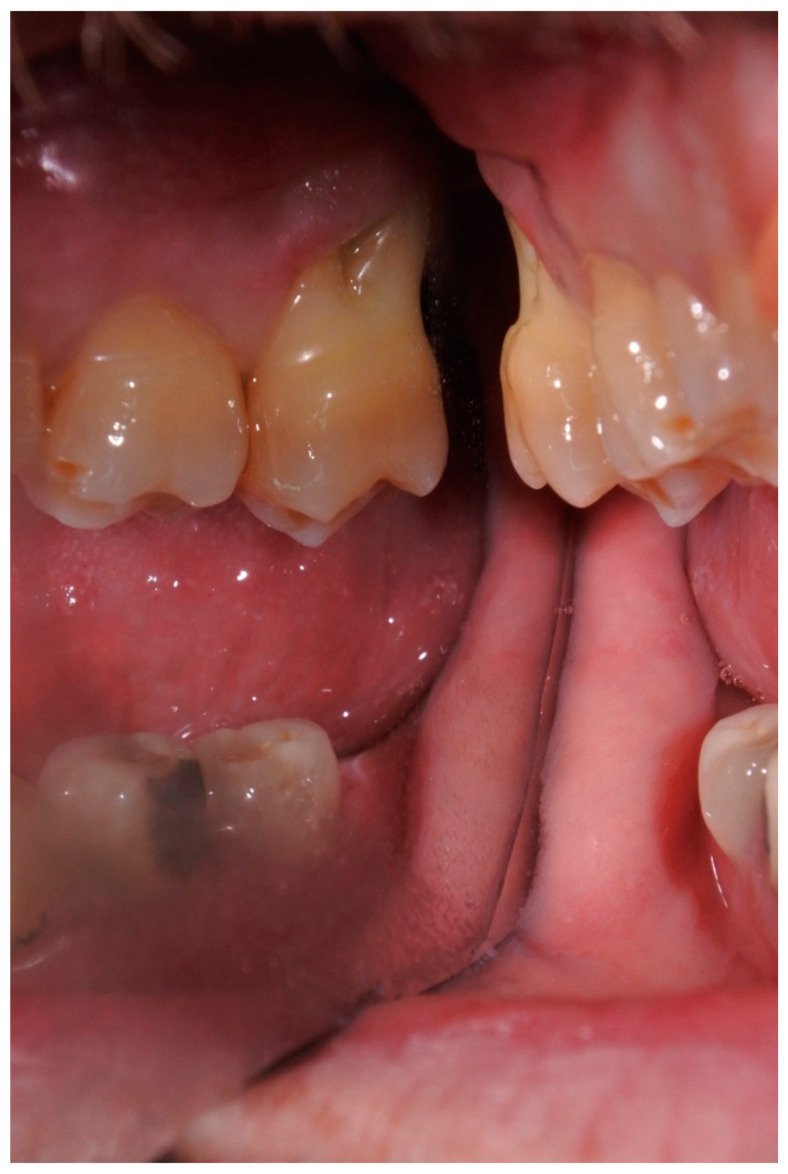
Intraoral photograph of tooth 1.7. with complete furcal exposure.

**Figure 2:**
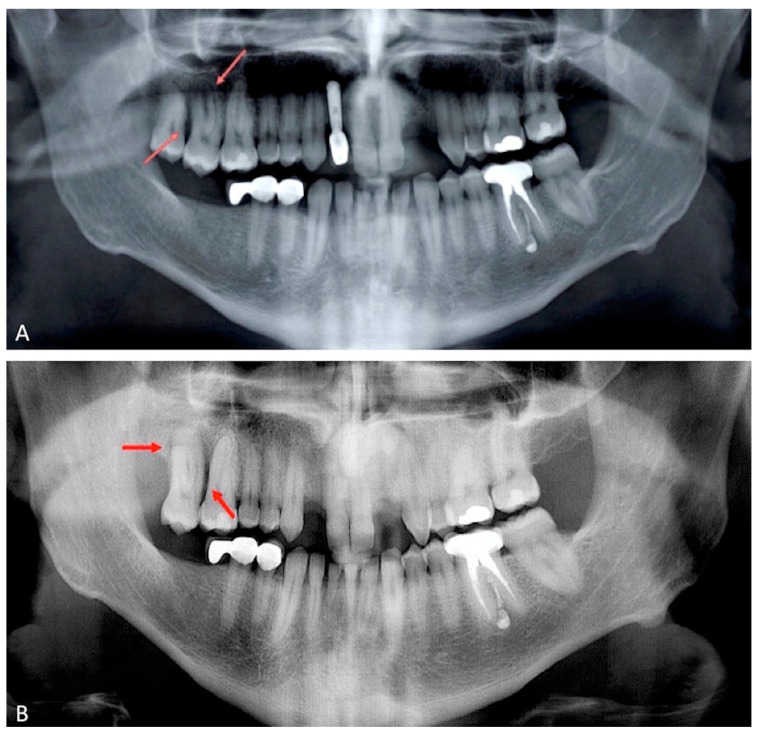
Orthopantomography of 2018; bone defects were not observed between 1.6. and 1.7. (panel **A**). Orthopantomography repeated in 2021. A severe horizontal bone loss and absence of tooth decay were observed. A horizontal radiolucent line in the third apical root was present (panel **B**). Red arrows indicate the lesions.

Extraoral examination showed non-palpable lymph nodes and no facial swelling. Intraoral examination showed a lack of some teeth, the presence of tooth-fixed prosthesis and conservative restorations. There was persistence of deciduous lower incisors with agenesis of permanent counterparts. In tooth 1.7., there was a severe gingival recession with a decreased attachment level (CAL) on the vestibular and distal surface (mesiovestibular = 5 mm; middle vestibular = 6 mm; distovestibular = 7 mm), with complete furcal exposure, grade 2 mobility and pain on percussion testing (Figure 1). A thermal vitality test did not elicit responses. All the other teeth were painless on percussion testing. A periodontal probing showed, over a gingival recession of 5–7 mm from the cementum–enamel junction, a pathologic probing depth (PD) on the vestibular and distal surface (mesiovestibular = 5 mm; middle vestibular = 5 mm; distovestibular = 4 mm). Probing was associated with pain and bleeding. The radiographic exam showed a severe horizontal bone loss and absence of tooth decay. A horizontal radiolucent line in the third apical root was also observed (Figure 2A). According to the new AAP classification of periodontal diseases, the presumptive diagnosis was of endo-periodontal lesion with root damage Grade 3. Examining the old radiography, a deep interproximal bone defect between 1.8. and 1.7. was observable, but no bone defects were observed between 1.7. and 1.6., and the radiolucent horizontal root line was absent (Figure 2B).

### 2.2. Surgical Treatment

Extraction of tooth 1.7. was planned two days after the first visit because the tooth was periodontally severely compromised and symptomatology was worsening, hindering any periodontal or endodontic treatment [20]. Non-surgical periodontal treatment with ultrasonic tips to remove dental biofilms and pre-operative mouthwash with chlorhexidine 0.20% for 60 s to reduce bacterial load were applied, and the tooth was anesthetized locally with articaine and adrenaline 1:100,000.

Sindesmotomy and luxation were performed with a rounded periosteal elevator; extraction was gently performed with dental forceps. An accurate alveolar toilette was performed with mechanical debridement of granulation tissue and subsequent intra-alveolar irrigation with sterile saline solution. A resorbable collagen sponge was positioned in the dental alveolus, and a criss-cross non-resorbable suture was performed to favor hemostasis. Analgesic pharmacological therapy was suggested.

**Figure 3:**
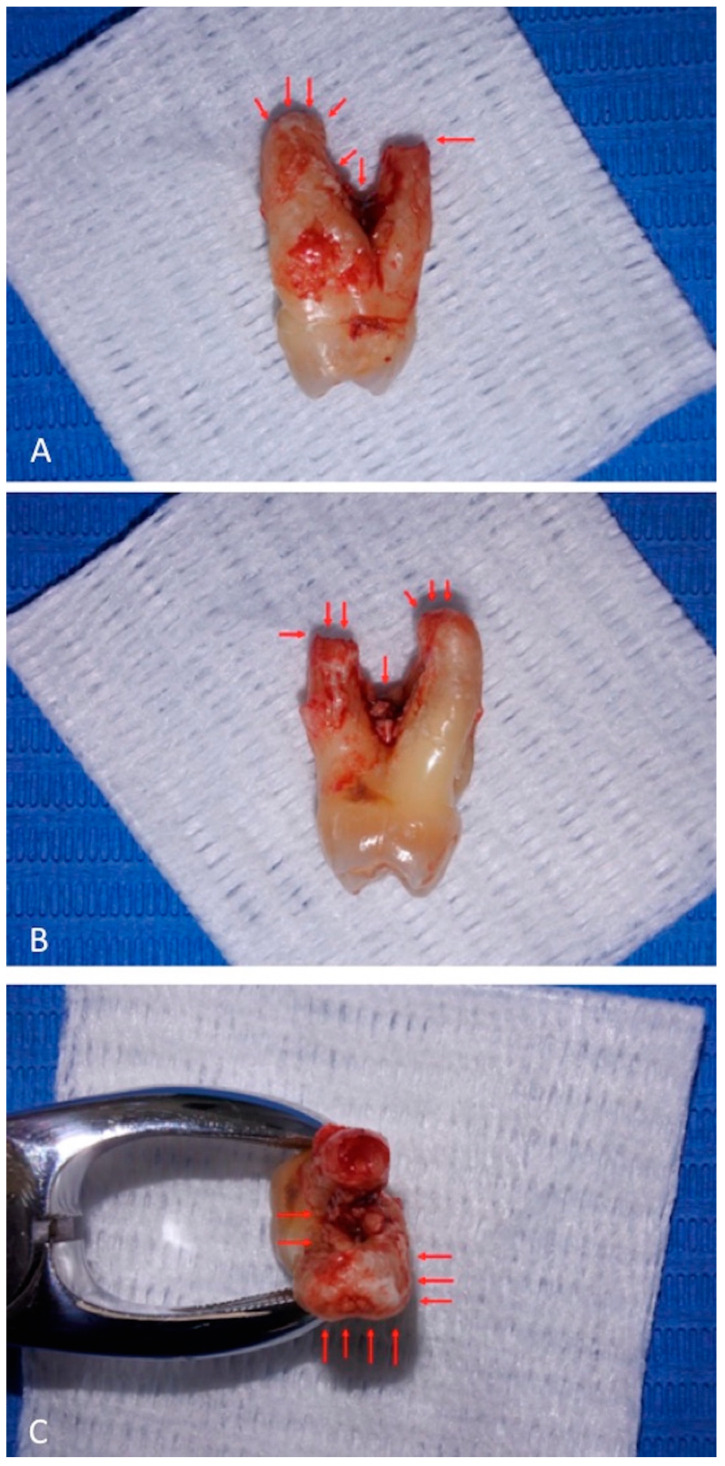
Decay or leakages are not appreciable on tooth crown surface. Root surfaces present adherent granulation tissue. The palatal root, on the right (panel **A**) and on the left (panel **B**) views, presents apical horizontal resorption. Root furcation shows adherent granulation tissue and biofilms. On the top view (panel **C**), apical resorption of the palatal root is evident (upper side), and adherent granulation tissue reaches the apical region of the vestibular root (lower side). Red arrows indicate the lesions.

At visual inspection, the tooth did not present deep decays or coronal leakages that could account for pulpal necrosis. Furthermore, the roots presented adherent granulation tissue and dental biofilms; in the apical portion of the palatal root, there was horizontal resorption that was visible as a radiolucent line at radiographic observation (Figure 3).

After 7 days, intraoral examination showed good wound healing and absence of signs and symptoms.

### 2.3. Sample Preparation

The crown was disinfected with 2.5% NaOCl (Niclor 2.5, Ogna, Maggiò, Italy) for 30 s. Preparation of the access cavity was carried out using a sterile high-speed diamond bur under sterile saline solution. All procedures were performed aseptically.

After preparation of the access cavity and pulpal exposure of tooth 1.7., bleeding was not observed, and the tissues did not appear vital throughout the cavity. The working length was determined, in both the palatal and vestibular canals, with K-File 10 (Dentsply-Maillefer, Ballaigues, Switzerland). After gentle irrigation with sterile saline solution, paper points #15 (Dentsply-Maillefer, Ballaigues, Switzerland) were positioned as deep as possible in the canals for 60 s, collected in sterile tubes (Eppendorf AG, Hamburg, Germany) and subsequently stored at −80 °C until use [17].

### 2.4. DNA Extraction and Quantification from Teeth Homogenates

After preparation of 10% homogenates in DMEM medium (Dulbecco’s Eagle Medium, Sigma–Aldrich, Missouri, USA), nucleic acids (DNA and RNA) were extracted using the commercial kit Cador Pathogen Minikit (Qiagen, Hilden, Germany). Briefly, 200 µL of the homogenates were mixed with the AVL buffer containing SDS and proteinase K and incubated for 15 min. Subsequently, the ACB buffer was added to the lysate, loaded into the silica columns and centrifuged at 8000 rpm for 1 min. The columns were washed with buffers AW1 and AW2 centrifuging at 8000 rpm for 1 min. After a vacuum centrifugation at 13,000 rpm for 1 min, the nucleic acids were eluted with AVE buffer by centrifugation at 13,000 rpm for 2 min. The nucleic acids were stored at −80 °C until further use.

DNA purity and concentrations were estimated using a NanoDrop spectrophotometer (Thermo Scientific, Waltham, MA, USA).

### 2.5. Bacterial 16S rRNA Gene Sequence Amplification, PCR Cleanup, Quantification and Next-Generation Sequencing

The 16S ribosomal RNA gene (16S rRNA) was the target gene used for metagenomic analysis. The specific targets used for the amplification were the V3 and V4 variable regions of 16S rRNA, which produce amplicons of about 460 bp in size [21]. The library was prepared according to a protocol described by the manufacturer (16S Metagenomic Sequencing Library Preparation-Illumina, San Diego, CA, USA). Briefly, the reaction was carried out in 25 µL volumes containing 12.5 ng of DNA sample, 0.2 µM of each primer and 12.5 µL of KAPA HiFi HotStart ReadyMix (Roche KAPA Biosystems; Boston, MA, USA). The following PCR conditions were used: initial denaturation at 95° for 3 min, followed by 25 cycles consisting of denaturation (95 °C for 30 s), annealing (55 °C for 30 s) and extension (72 °C for 30 s), and a final extension step at 72 °C for 5 s. PCR products were visualized on QIAxcel Advanced System (Qiagen, Hilden, Germany). Each PCR product was purified used a magnetic bead capture by AMPure XP beads (Beckman Coulter, Brea, CA, USA), according to the manufacturer’s instructions (Illumina, San Diego, CA, USA). Then, for each amplicon, the dual indices were ligated (Index i5 and i7 Illumina) using the XT DNA Library Preparation Kit (Illumina, San Diego, CA, USA), followed by a new PCR cleanup with AMPure XP beads. The final libraries were visualized using QIAxcel Advanced System (Qiagen, Hilden, Germany) to verify the size of the bands and were quantified using a fluorometric method (Qubit 2.0, Invitrogen, Carlsbad, CA, USA). The libraries were pooled in an equimolar concentration, and the final concentration of the libraries (4 nm) was determined.

Sequencing was performed using MiSeq (Illumina, San Diego, CA, USA) at 2 × 250 bp, in paired-end mode. Sequencing data were processed with the DADA2 package (v1.18) [22] available through the R programming language, following the recommendations available at https://benjjneb.github.io/dada2/tutorial.html (accessed on 19 May 2021). The DADA2 workflow outputs an amplicon sequence variant (ASV) table, which represents an inference of sequences (and their related abundance) from the sample under investigation. Taxonomic assignment of ASVs was performed at the genus level with the naive Bayesian classifier method as implemented in DADA2 [23], using the Silva (v138) training dataset [24] as a reference database. Taxonomic assignment of ASVs at the species level was performed using the exact matching method, using an ad hoc Silva [25] training dataset [24]. Taxonomic abundance was plotted with the R package ggplot2 [26].

## 3. Results

**Figure 4:**
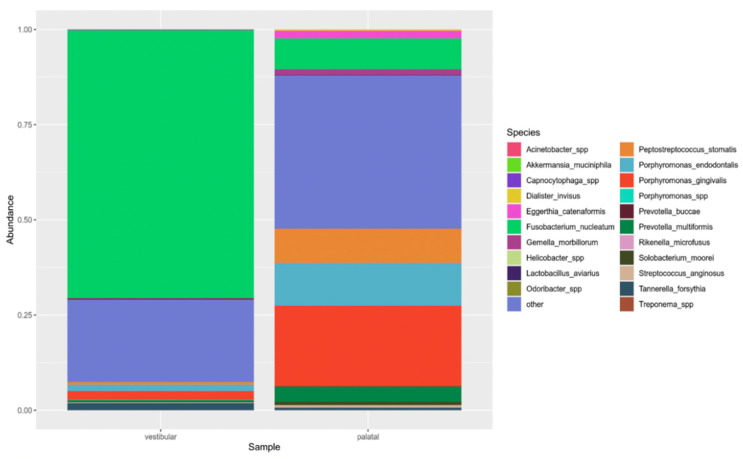
Relative abundance of taxa in the vestibular and palatal samples. The top 15 species and genera are shown.

By NGS analysis of 16S rRNA, a different microbiological pattern was found in the vestibular and palatal canals of the patient. In particular, in the vestibular site, *Fusobacterium nucleatum* was identified as the predominant species (70% of reads). Other detected taxa were anaerobic bacteria, including members of the phylum *Bacteroidia* (notably, *Porphyromonas* species such as *P. gingivalis* and *P. endodontalis* represented by 4% of total reads), the genus *Treponema* (10%) and the class *Clostridia* (3%) (Figure 4).

In the palatal canal, there was not a predominant taxon. Several anaerobic taxa were identified, such as *F. nucleatum* (8%), and two species belonging to *Porphyromonas spp.* (*P. gingivalis* and *P. endodontis*, with 21% and 11% of total reads, respectively); furthermore, *Parvimonas spp.* (13%), *Peptostreptococcus stomatis* (9%) and *Prevotella multiformis* (8%) were detected (Figure 4).

## 4. Discussion

This case report presented an unusual EPL, probably developed in the arch over a few years and characterized by a different distribution and subdivision of bacterial species in the endodontic system. Due to its grading, aggressivity and poor prognosis, it was decided to proceed with tooth extraction. Pulpal necrosis was likely caused by a primary deep periodontal pocket that affected the apical foramen lacking a pulpal bloodstream, and, subsequently, the endodontic compartment was infected by bacteria originated from the periodontal pocket through the apical foramen, the accessory canals and/or dentinal tubules, as described in the literature [10,12,15]. In bacterial infections, tissue damage and progression of endo-periodontal disease can be determined and sustained by host-released inflammatory mediators. In particular, Gal-3 [6] is implicated in pro-inflammatory stimuli in innate immune cells [27], such as neutrophils [28]. A significant higher expression of Gal-3 is reported in inflammatory diseases such as periapical granulomas, radicular cysts, osteonecrosis of the jaw associated with bisphosphonates and periodontal diseases [29]. Additionally, Gal-3 activates the NLRP3 inflammasome [7,8], capable of converting pro-IL-1β to IL-1β, an important inflammatory mediator that participates in inflammatory processes and is implicated in periodontal tissue destruction [30]. Moreover, high expression of NLRP3 and IL-1β has been found in human gingival tissues with severe chronic periodontitis [31]. NLRP3 is highly expressed in monocytes/macrophages [32] and influences their differentiation in osteoclasts [31], with induction of bone resorption.

The absence of coronal decay was useful to rule out the entrance of bacteria from the pulpal chamber, suggesting the potential of periodontal bacteria to invade the endodontic space. In fact, EPLs without coronal decay or leakages are rarely observed, and it is not possible to understand whether endodontic bacteria originated from the periodontal compartment or from the deep decay

The endodontic microbiome has been defined as a “melting pot”, characterized by polymicrobial complexes [33]. In our analysis, we observed a clear separation of microbial complexes in the endodontic space.

A predominant bacterial population (*Fusobacterium nucleatum*) was observed in one endodontic canal, whist in the other canal, there were, at least, six major bacterial populations equally represented. It is possible to hypothesize that in the same tooth, there were various ecologic niches, competing with each other. This hypothesis could explain a common clinical dilemma that occurs when, in the same multirooted tooth, some canals present bleeding/purulent exudate, or when, on mechanical instrumentation analysis, some canals appear symptomatic whilst others do not. Overall, these common clinical aspects are partially explained by a different inflammation of the endodontic canals [34], probably linked to different bacterial populations causing different symptoms.

Different bacterial populations related to different clinical presentations can be discovered in periodontal diseases. Severe acute forms such as periodontal abscesses and necrotizing periodontal diseases seem to be characterized by different bacterial populations with respect to chronic periodontitis. In particular, in severe acute forms of periodontal disease, *Porphyromonas gingivalis*, *Prevotella intermedia, Prevotella melaninogenica, Fusobacterium nucleatum, Tannerella forsythia, Treponema* species, *Campylobacter* species, *Capnocytophaga* species and *Aggregatibacter actinomycetemcomitans* are common [12].

## 5. Conclusions

EPLs pose a challenge in tooth maintenance therapies, demonstrating the strict anatomical correlation between the endodontic and periodontal systems. The oral microbiome is incredibly complex, with 200 predominant bacterial species and 700 predominant taxa, with various interactions between different bacterial species and subsequent pathogenicity levels and clinical manifestations, yet it is under the influence of the inflammatory response and of inflammatory mediators released by the host immune system. As observed in this report, with a particular bacterial distribution in the endodontic system, it is important to gather information on the oral microbiome. New microbiological approaches based on metagenomic analysis or on characterization of universal diagnostic targets, such as the rRNA 16S for bacteria, are particularly useful, allowing the detection and characterization of uncultivatable microbiological species in biological samples and overcoming the limits of classical microbiological techniques.

## Data Availability

The data presented in this study are available on request from the corresponding author.
